# Early detection of pre-malignant lesions in a KRAS^G12D^-driven mouse lung cancer model by monitoring circulating free DNA

**DOI:** 10.1242/dmm.036863

**Published:** 2019-02-12

**Authors:** Callum P. Rakhit, Ricky M. Trigg, John Le Quesne, Michael Kelly, Jacqueline A. Shaw, Catrin Pritchard, L. Miguel Martins

**Affiliations:** 1 MRC Toxicology Unit, University of Cambridge, Lancaster Road, Leicester LE1 9HN, UK; 2 Leicester Cancer Research Centre, University of Leicester, Leicester LE2 7LX, UK; 3 Core Biotechnology Services, University of Leicester, Leicester LE1 7RH, UK

**Keywords:** KRAS^G12D^, Mouse model, Circulating free DNA, cfDNA, Early detection, Lung adenocarcinoma

## Abstract

Lung cancer is the leading cause of cancer-related death. Two-thirds of cases are diagnosed at an advanced stage that is refractory to curative treatment. Therefore, strategies for the early detection of lung cancer are urgently sought. Total circulating free DNA (cfDNA) and tumour-derived circulating tumour DNA (ctDNA) are emerging as important biomarkers within a ‘liquid biopsy’ for monitoring human disease progression and response to therapy. Owing to the late clinical diagnosis of lung adenocarcinoma, the potential for cfDNA and ctDNA as early detection biomarkers remains unexplored. Here, using a Cre-regulated genetically engineered mouse model of lung adenocarcinoma development, driven by Kras^G12D^ (the *Kras^LSL-G12D^* mouse), we serially tracked the release of cfDNA/ctDNA and compared this with tumour burden as determined by micro-computed tomography (CT). To monitor ctDNA, a droplet digital PCR assay was developed to permit discrimination of the *Kras^Lox-G12D^* allele from the *Kras^LSL-G12D^* and *Kras^WT^* alleles. We show that micro-CT correlates with endpoint histology and is able to detect pre-malignant tumours with a combined volume larger than 7 mm^3^. Changes in cfDNA/ctDNA levels correlate with micro-CT measurements in longitudinal sampling and are able to monitor the emergence of lesions before the adenoma-adenocarcinoma transition. Potentially, this work has implications for the early detection of human lung adenocarcinoma using ctDNA/cfDNA profiling.

A video abstract for this article is available at https://youtu.be/Ku8xJJyGs3U.

This article has an associated First Person interview with the joint first authors of the paper.

## Abstract (precis)

**Summary:** A liquid biopsy approach is capable of tracking early lung cancer lesions, before the adenoma-adenocarcinoma transition, in an early lung cancer mouse model driven by a *KRAS* mutation.

## INTRODUCTION

Circulating free DNA (cfDNA) was first identified in the human bloodstream during the first half of the 20th century (Mandel and Metais, 1948), although only in the late 1980s was cfDNA isolated from the plasma of cancer patients, shown to be partially derived from tumours (Stroun et al., 1987), representing the so-called circulating tumour DNA (ctDNA) fraction. ctDNA is a reliable biomarker for identifying oncogenic changes within the body (Schwarzenbach et al., 2011) and has implications for both the early detection and monitoring of cancer. Changes in the molecular profile of ctDNA can be used to detect early-stage cancer lesions (Cheng et al., 2017), classify the molecular profiles of existing tumours (Burrell et al., 2013), identify the emergence of resistance (Diaz et al., 2012) and track the evolution of cancer genomes in response to targeted drug therapies (Abbosh et al., 2017). In this regard, ctDNA analysis is transforming the monitoring of cancer after diagnosis and has been established as a prognostic factor for lung cancer patients (Cabral et al., 2010; Goebel et al., 2005).

Monitoring a patient's ctDNA profile is less invasive than tissue biopsy, with the reduced stress and cost allowing for more frequent sampling. Repeat sampling allows for more targeted, personalised therapy in the face of tumour evolution. ctDNA may also have the potential to enable the discovery of newly emergent cancers, undetectable by imaging or other diagnostic procedures (Abbosh et al., 2017; Chaudhuri et al., 2017; Garcia-Murillas et al., 2015). Accordingly, ctDNA profiling approaches are being developed to improve the monitoring and characterisation of residual disease in lung cancer, which could help improve outcomes in the adjuvant disease setting (Abbosh et al., 2017). However, the genetic profiling of ctDNA in individuals with early disease lesions has been limited, due to inherent challenges in building a cohort of study patients with identifiable pre-malignant lesions.

Gain-of-function mutations in *KRAS* are present in ∼25% of human lung adenocarcinomas and are truncal events, acquired early in disease development (Abbosh et al., 2017; Rakhit et al., 2017). Oncogenic mutants of *KRAS*, such as the prevalent *KRAS^G12D^* mutation, have transforming activity and are thought to be founder mutations as they can initiate and drive tumour progression in mouse models (Guerra et al., 2003; Jackson et al., 2001; Sutherland et al., 2014). The autochthonous *Kras^LSL-G12D^* conditional mouse knock-in model, which allows for endogenous expression of Kras^G12D^ following Cre induction, has been used extensively to study the mechanisms underpinning early disease initiation and maintenance (Jackson et al., 2005; Jackson et al., 2001; Sheridan and Downward, 2015; Sutherland et al., 2014). Evidence shows that the model recapitulates early-stage lung adenocarcinoma development, through the formation of atypical adenomatous hyperplasia (AAH), epithelial hyperplasia of the bronchioles and papillary adenomas (Jackson et al., 2001; Nikitin et al., 2004). Early-stage lung adenocarcinomas are occasionally seen at late stages, following prolonged Kras^G12D^ expression, and can be accelerated by a combined *p53* (also known as *Trp53*) mutation (Jackson et al., 2005).

Here, we use the *Kras^LSL-G12D^* mouse model to explore the utility of both total cfDNA levels and ctDNA as an early-stage biomarker. We show that cfDNA/ctDNA is detectable in mice bearing pre-malignant lung lesions, prior to the adenocarcinoma transition, and we correlate the liquid biopsy data with the emergence of pre-malignant lung lesions as detected by longitudinal micro-computed tomography (CT).

## RESULTS

### Comparing tumour size and number in *Kras^+/Lox-G12D^* mice using micro-CT and histology

**Fig. 1.**
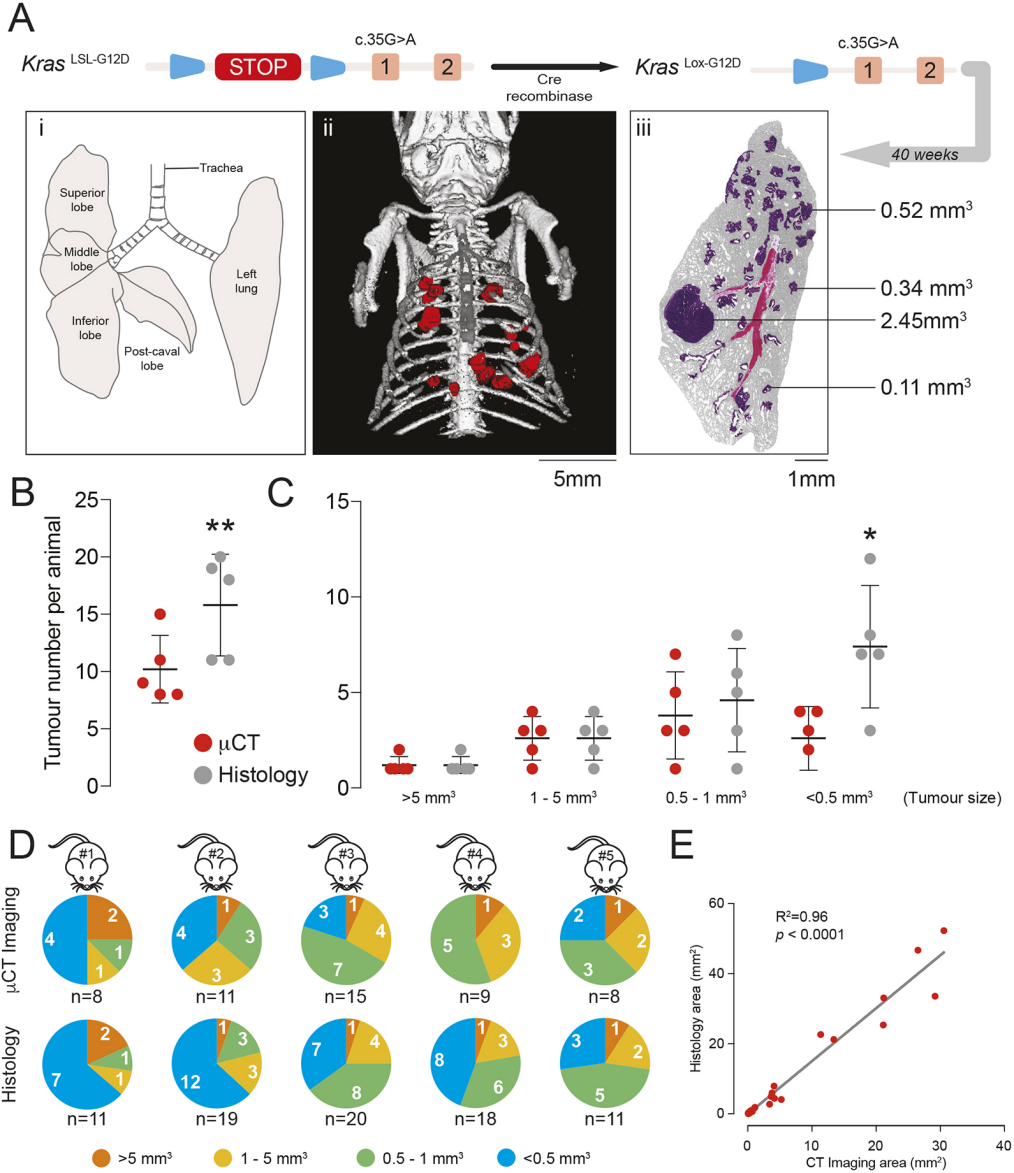
**Comparison of micro-CT imaging and histological analysis of tumours for measuring tumour burden.** (A) Schematic of the *Kras^LSL-G12D^* allele. Expression of Cre recombinase allows for removal of the LoxP-STOP-LoxP cassette and formation of the *Kras^Lox-G12D^* allele expressing Kras^G12D^. The anatomy of the mouse lung is shown (i). Tumour number and size were estimated at the endpoint by micro-CT (ii, tumours in red) or histology (iii) following 40 weeks of induction of mice with Ad5-mSPC-Cre. The histology image shows tumours with different volumes. (B) Comparison of the total number of tumours detected by micro-CT and histology in five individual mice at the endpoint of 40 weeks p.i. with Ad5-mSPC-Cre. There were significantly more tumours (*P*<0.01) detected by histological analysis than by micro-CT (mean±s.d.; **P<0.01, paired Student's *t*-test). (C) Comparison of the total number of tumours at endpoint (40 weeks p.i. with Ad5-mSPC-Cre), detected by micro-CT and histology, grouped (binned) according to size, in five individual animals (mean±s.d.; *P<0.05, paired Student's *t*-test). (D) Size distribution of tumours detected by micro-CT (top row) or histology (bottom row) in five individual mice at endpoint (40 weeks p.i. with Ad5-mSPC-Cre). The total number of tumours identified by the two methods in each animal (n) is indicated. (E) Comparison of tumour volumes as determined by micro-CT and serial sectioning of the entire tumour, H&E staining and tumour area quantitation. The two methods showed a linear correlation.

To induce expression of KRas^G12D^ in the mouse lung, adenoviral-Cre vectors are delivered to *Kras^+/LSL-G12D^* mice using intranasal delivery (Fig. 1A), and the most commonly used viruses used for this purpose are either Ad5-CMV-Cre or Ad5-mSPC-Cre (Jackson et al., 2001; Sutherland et al., 2014). With Ad5-CMV-Cre, the Cre recombinase is expressed from the ubiquitous cytomegalovirus (CMV) promoter and generates a range of lung pathologies including AAH/adenoma and bronchial hyperplasia (BH) (Jackson et al., 2001). Ad5-CMV-Cre is also known to induce recombination of the *Kras^LSL-G12D^* allele in lung resident myeloid cells as well as features of the Langerhans cell histiocytosis phenotype (Kamata et al., 2017). In the case of Ad5-mSPC-Cre, the Cre recombinase is expressed from the mouse surfactant protein C (SPC; also known as Sftpc) promoter, allowing more restricted expression of Kras^G12D^ to alveolar type II cells and inducing the development of AAH/adenoma (Sutherland et al., 2014). With both models, overt adenocarcinomas are detectable at a low frequency over prolonged periods (Jackson et al., 2001; Sutherland et al., 2014). A previous study has shown a good correlation between micro-CT and histology using the *Kras^+/LSL-G12D^* model infected with Ad5-CMV-Cre. Therefore, we focused on extending this analysis to *Kras^+/LSL-G12D^* mice infected with Ad5-mSPC-Cre.

We examined the dynamic range for tumour detection in *Kras^+/LSL-G12D^* mice at 40 weeks postinfection (p.i.) with Ad5-mSPC-Cre by comparing the tumour burden at endpoint using histology and micro-CT (Fig. 1A). The lesions detected at this endpoint were heterogeneous in size (Fig. 1A, iii). We found that the number of tumours observed by histology was significantly higher (*P*<0.01) than that observed when micro-CT was used (Fig. 1B). A further analysis, in which tumours were grouped according to volume using data binning, revealed that the sensitivity of micro-CT for tumour detection was lost when tumour volume fell below 0.5 mm^3^, with micro-CT showing a significantly smaller (*P*<0.05) tumour number (Fig. 1C,D).

To ensure that micro-CT provides an accurate indication of individual tumour volume for tumours with volumes above 0.5 mm^3^, we compared results from micro-CT with those from tumour area quantitation determined by serial sectioning followed by Haematoxylin and Eosin (H&E) staining. The two methods showed a linear correlation (Fig. 1E), indicating that both methods were concordant for tumour detection above a 0.5-mm^3^ threshold. Overall, our data show that micro-CT provides an accurate value for tumour volumes above a lower limit of 0.5 mm^3^, consistent with data from Kirsch and colleagues (Kirsch et al., 2010).

### Analysis of tumour histology types

**Fig. 2.**
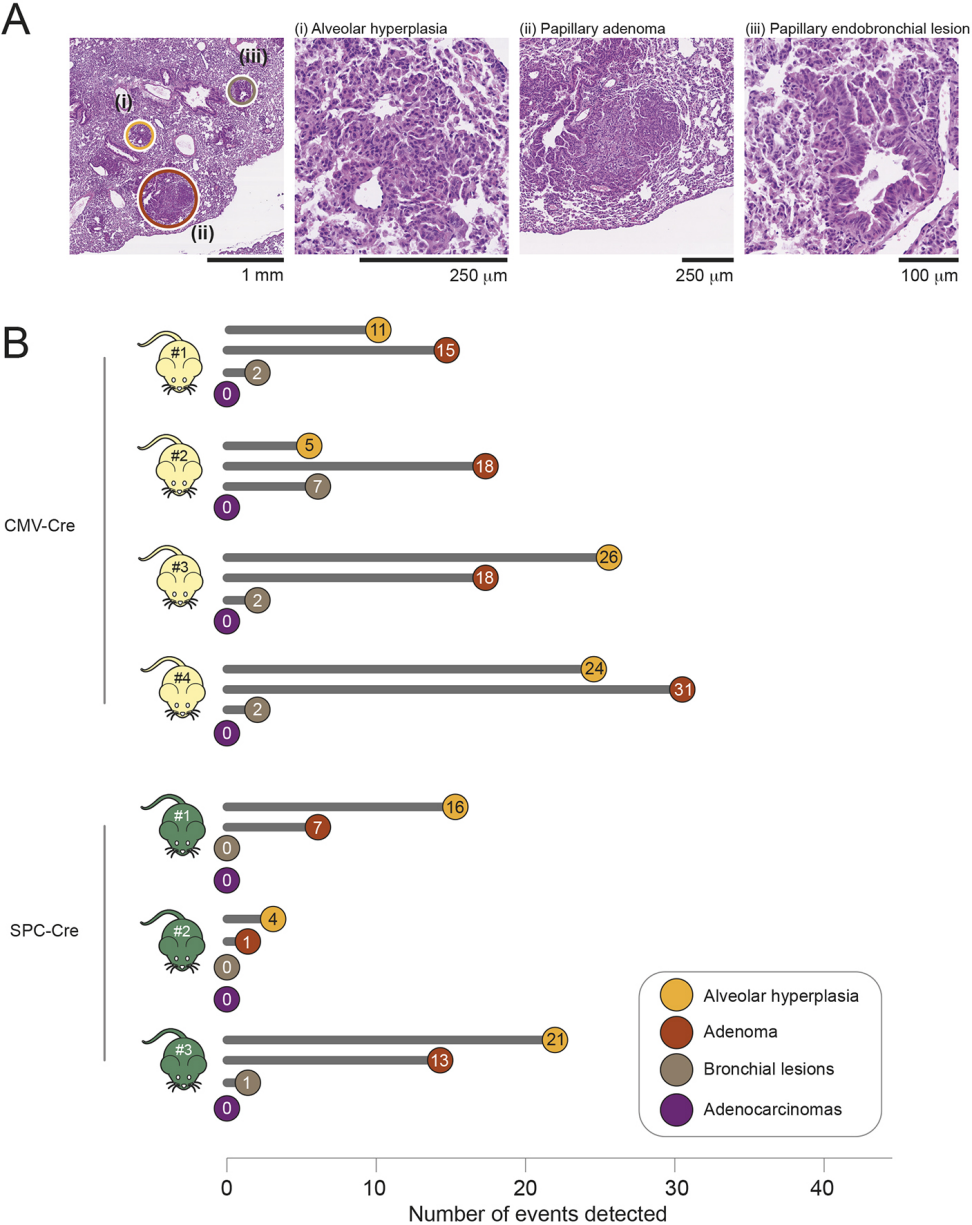
**Histological assessment of pathological alterations in mice expressing the *Kras^Lox-G12D^* allele.** (A) Representative H&E-stained lung sections of *Kras^+/LSL-G12D^* mice infected with Ad5-CMV-Cre expression and analysed 20 weeks p.i. The different pathologies detected in the far left image are magnified in images i-iii, showing representative examples of alveolar hyperplasia (i), papillary adenoma (ii) and papillary endobronchial lesions (iii). (B) Quantitative assessment of each pathology detected in H&E-stained sections of lung tissue collected from *Kras^+/LSL-G12D^* mice infected with either Ad5-CMV-Cre (n 4) or Ad5-mSPC-Cre (*n*=3) at endpoint (20 and 40 weeks, respectively).

To gain an assessment of the spectrum of lung tumour histology types, we undertook histopathological evaluation of H&E-stained lung sections from *Kras^+/LSL-G12D^* mice at 20 weeks p.i. with Ad5-CMV-Cre, and at 40 weeks p.i. induction with Ad5-mSPC-Cre, using the recommended criteria of mouse lung tumour classification (Nikitin et al., 2004). Fig. 2A provides representative images of H&E-stained sections of AAH, adenoma and bronchial lesions in Ad5-CMV-Cre-infected mice. As previously reported (Jackson et al., 2001; Sutherland et al., 2014), our analysis showed the presence of AAH and adenomas in both sets of mice. Adenomas were mostly of simple papillary pattern but rare small foci of solid growth were identified. Bronchial lesions were evident in Ad5-CMV-Cre-infected mice, as expected, but were largely absent from Ad5-mSPC-Cre-infected mice (Fig. 2B). Lesions of each histological type were counted (Fig. 2B), and this showed more AAHs/adenomas in the Ad5-CMV-Cre-infected mice compared with Ad5-mSPC-Cre-infected mice. However, there was a high degree of inter-mouse variability, regardless of the type of virus used, for reasons that are likely related to some variability in the amount of virus inhaled by individual mice. Interestingly, no overt adenocarcinomas were observed in either the Ad5-CMV-Cre- or Ad5-mSPC-Cre-infected *Kras^+/LSL-G12D^* mice at the time points analysed.

### *In vivo* monitoring of tumour burden by measuring levels of total cfDNA

**Fig. 3.**
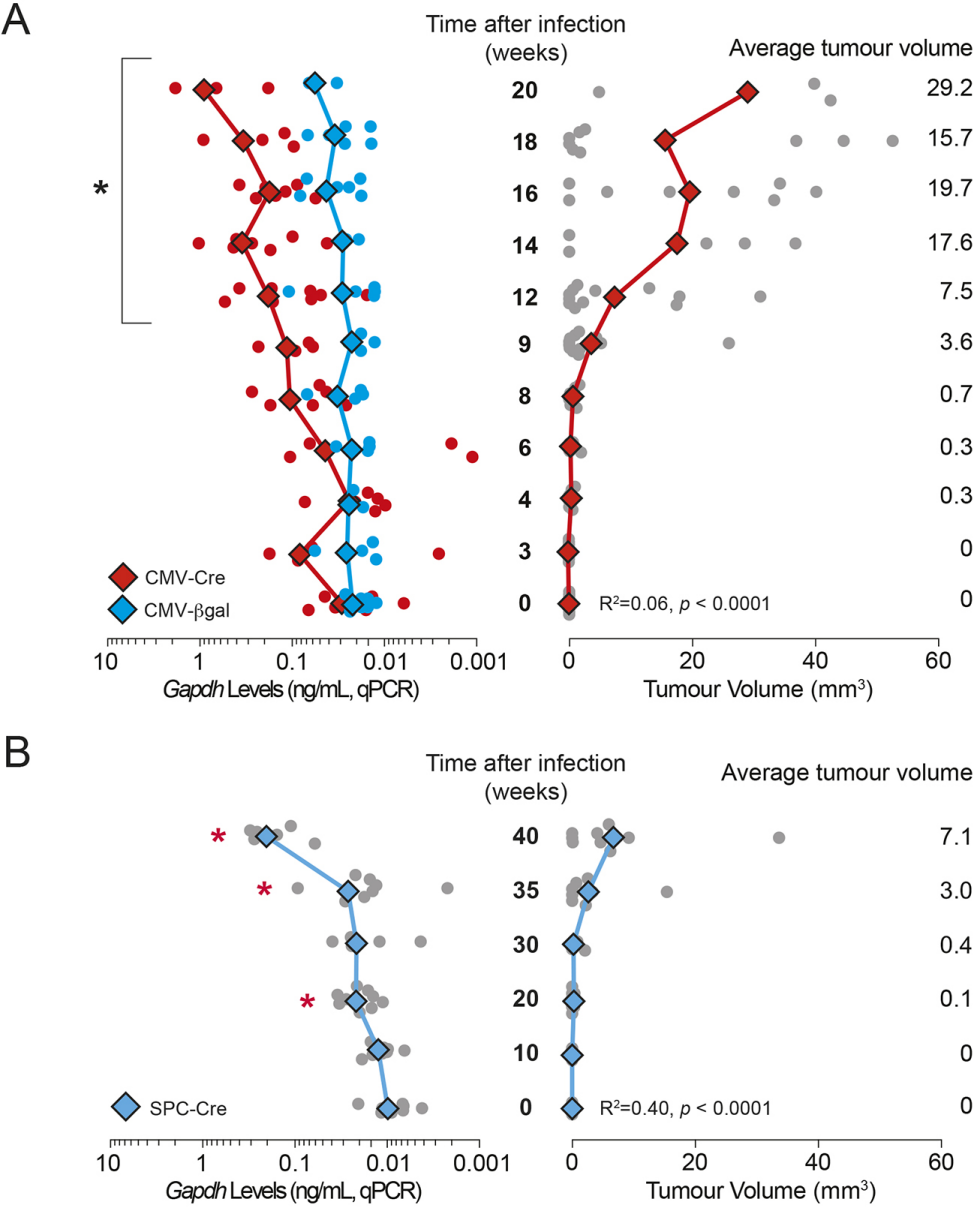
**Monitoring of cfDNA levels using *Gapdh* analysis by qPCR.** (A) *Gapdh* levels as measured by qPCR in cfDNA in comparison to tumour burden in *Kras^+/LSL-G12D^* mice over a time course following infection with Ad5-CMV-Cre (red) or Ad5-CMV-βgal (blue). Total tumour burden was quantitated from micro-CT imaging. *Gapdh* levels were measured by qPCR of circulating DNA derived from the plasma of mice at each time point. Mean values are indicated by diamonds/lines; values for individual mice are indicated by circles (*n*=3-12 at each time point). *P<0.05 for two-tailed unpaired Student's *t*-test comparisons between mean values at a given time point and values at time=0. The correlation coefficients between *Gapdh* levels and tumour burden are indicated (linear regression analysis; *P*-value, goodness of fit). (B) *Gapdh* levels in cfDNA as measured by qPCR in comparison to tumour burden in *Kras^+/LSL-G12D^* mice over a time course following infection with Ad5-mSPC-Cre. Mean values are indicated by diamonds/lines; values for individual mice are indicated by circles (*n*=4-9 at each time point). *P<0.05 for two-tailed unpaired Student's *t*-test comparisons between mean values at a given time point and values at time=0. The correlation coefficients between *Gapdh* levels and tumour burden are indicated (linear regression analysis; *P*-value, goodness of fit).

We next assessed whether non-invasive monitoring of lung tumour development could be achieved by profiling levels of total cfDNA. We infected *Kras^+/LSL-G12D^* mice with viruses carrying either Ad5-CMV-Cre or Ad5-CMV-βgal (control) for up to 20 weeks (Fig. 3A), or with Ad5-mSPC-Cre for up to 40 weeks (Fig. 3B), by intranasal delivery, and monitored tumour development every 2 weeks in the case of Ad5-CMV-Cre/Ad5-CMV-βgal, and every 5-10 weeks in the case of Ad5-mSPC-Cre, by micro-CT scanning (Fig. 3A,B, right panels). We collected blood samples for cfDNA analysis, in parallel with the micro-CT imaging.

We observed a more rapid increase in tumour volume with Ad5-CMV-Cre infection (Fig. 3A) than with Ad5-mSPC-Cre (Fig. 3B) infection, which was consistent with previously reported data (Jackson et al., 2001; Sutherland et al., 2014) and the data shown in Fig. 2B. In Ad5-CMV-Cre-infected mice, tumours larger than 0.5 mm^3^ were first detectable by micro-CT at ∼9 weeks p.i. (Fig. 3A), whereas in Ad5-mSPC-Cre-infected mice, tumours above this threshold were detectable beginning at 35 weeks p.i. (Fig. 3B).

To assess total cfDNA levels in blood, a quantitative real-time PCR (qPCR) assay targeting a 113-bp single-copy locus within the *Gapdh* genomic region was used. This assay has a dynamic range of at least 500-fold and can detect the equivalent of one copy (3.3 pg; one haploid genome equivalent) of *Gapdh* (R. M. Trigg, Molecular analysis of circulating cell-free DNA in lung cancer, PhD thesis, University of Leicester, 2017). As expected, given the lower tumour burden of the Ad5-mSPC-Cre mice, the cfDNA levels induced by Ad5-mSPC-Cre were lower than those induced by Ad5-CMV-Cre (Fig. 3A,B, left panels). However, for tumours of equivalent volumes, cfDNA levels in Ad5-CMV-Cre- and Ad5-mSPC-Cre-infected mice were broadly similar, suggesting that Kras^G12D^ expression in lung resident myeloid cells or BH induced by Ad5-CMV-Cre had little effect on overall cfDNA release.

A significant increase in cfDNA levels was first detected at 12 weeks p.i. in the case of Ad5-CMV-Cre (Fig. 3A) in comparison to samples at baseline. However, in the case of Ad5-mSPC-Cre, a significant increase in cfDNA levels was first detectable at 20 weeks p.i. (Fig. 3B), but this was not maintained until after 35 weeks p.i. For Ad5-CMV-βgal, cfDNA levels remained consistently low throughout the time course (Fig. 3A). These data show that overall cfDNA levels were increased in mice bearing lesions that were representative of early pre-malignant lung lesions, without transition to adenocarcinoma, compared with healthy controls. For both adenoviral systems, cfDNA levels, as measured by qPCR analysis, did not reproducibly improve the threshold for tumour burden compared with micro-CT scanning, although the data for the 20-week time point in Ad5-mSPC-Cre mice potentially suggests that a more sensitive cfDNA assay method could facilitate this.

### Development of a PCR assay to monitor ctDNA through *Kras^Lox-G12D^*

Although cfDNA levels can give an indication of tumour burden, their assessment is not able to distinguish tumour-derived ctDNA from that derived from apoptosis of other healthy cells. Therefore, we developed an assay for measuring ctDNA through the detection of the tumour-derived *Kras^Lox-G12D^* allele in blood (see Methods in the supplementary material). Initial studies using endpoint PCR approaches detected the presence of this allele in the cfDNA of Ad5-CMV-Cre-infected *Kras^+/LSL-G12D^* mice at 12 weeks p.i. (R. M. Trigg, Molecular analysis of circulating cell-free DNA in lung cancer, PhD thesis, University of Leicester, 2017), encouraging the development of a qPCR assay.

An assay that specifically detects the *Kras^Lox-G12D^* allele was required, but this proved challenging due to similarities with the *Kras^LSL-G12D^* and *Kras^WT^* alleles. A unique 34-bp region that contains the single LoxP sequence and has 13-bp palindromic motifs flanking a central 8-bp spacer sequence was identified (Fig. S1A). This sequence was determined to have a propensity to form a stem-loop structure (Fig. S1B) that occludes access to primers and DNA polymerase (Huang et al., 2007). Moreover, this LoxP sequence is flanked by palindromic *Sal*1 restriction sites, which contribute a further 6 bp to the stem-loop structure; this 19-bp structure (Fig. S1C) has a predicted melting temperature (T_m_) of 65.5°C by *in silico* prediction using mFold (Zuker, 2003). To overcome the inhibitory effect of this stem-loop on PCR amplification, several strategies were attempted.

The first PCR strategy involved targeting the stem-loop with dual-labelled TaqMan probes incorporating a 3′ minor groove binder moiety (Fig. S2, MGB-1, MGB-2 or MGB-3). However, this failed to permit PCR amplification, even when destabilising agents such as betaine, urea and dimethyl sulfoxide were included (data not shown). A second strategy was taken in which a hydrolysis probe was designed to include ‘locked nucleic acid’ (LNA) bases, a chemical modification that increases both the T_m_ and binding specificity of the probe while also providing strand-invasion properties. This LNA probe, LNA-1 (Fig. S2), permitted qPCR amplification of the *Kras^Lox-G12D^* allele, but the efficiency was poor (∼85%; Fig. S3A,B). To attempt to improve the amplification efficiency, we next adapted the LNA-1 probe to a droplet digital PCR (ddPCR) assay. Although this approach provided some discrimination between positive and negative droplets, there was an additional clustering of droplets above the main negative cluster, above 5000 fluorescence units (Fig. S4), indicating the presence of artefacts caused by autofluorescence.

**Fig. 4.**
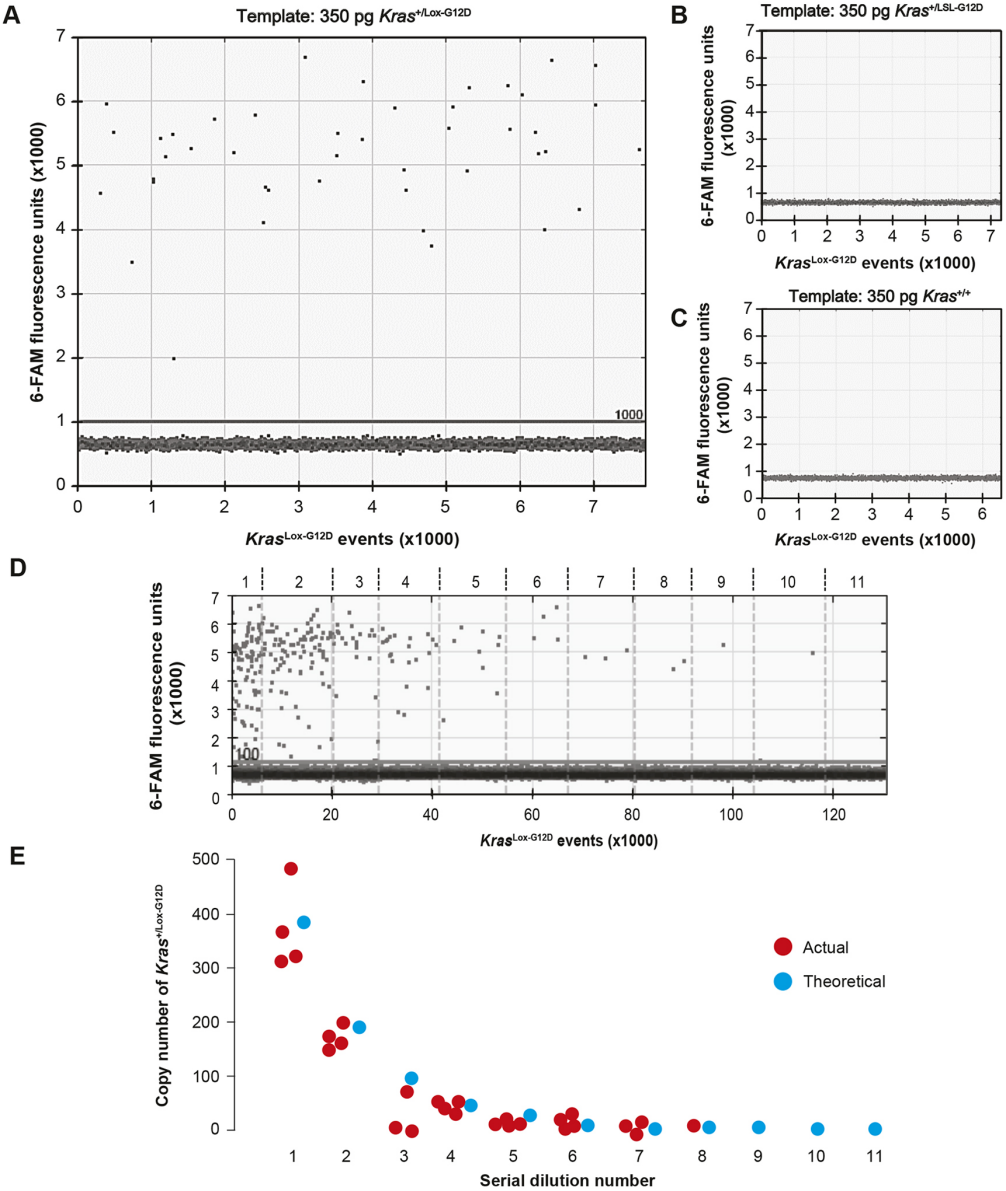
**Validation of ddPCR assay for detection of *Kras^Lox-G12D^* allele using LNA-2 primers.** (A-C) One-dimensional droplet plot of LNA-2 assay (comprising probe LNA-2 and primers flanking the recombined LoxP sequence in *Kras^Lox-G12D^*) with *Kras^+/Lox-G12D^* MEF genomic DNA (A), *Kras^+/LSL-G12D^* MEF genomic DNA (B) and *Kras^+/+^* MEF genomic DNA (C). Primers were annealed at 62°C, following a gradient PCR experiment to determine the optimum annealing temperature (T_a_, not shown). A manual threshold of 1000 fluorescence units was selected. (D) *Kras^+/Lox-G12D^* MEF genomic DNA was serially diluted into a background of *Kras^+/LSL-G12D^* MEF genomic DNA. A one-dimensional droplet plot for the 133-bp LNA-2 assay at each serial dilution is shown. A manual threshold of 1100 fluorescence units was selected. (E) Actual versus theoretical copy number of *Kras^Lox-G12D^* at each serial dilution.

A second LNA probe, LNA-2 (Fig. S2), was next designed to destabilise both stem-loops simultaneously, using a ‘double destabilisation’ strategy (Esposito et al., 2003). The combination of probe LNA-2 with the 133-bp amplicon primers provided good discrimination between positive and negative droplets with *Kras^+/Lox-G12D^* genomic DNA as a template (Fig. 4A-D). To determine the sensitivity of the LNA-2 assay, *Kras^+/Lox-G12D^* genomic DNA was spiked into a background of *Kras^+/LSL-G12D^* genomic DNA and serially diluted twofold over 11 points (Table S1). To ensure that the concentration of *Kras^LSL-G12D^* in each dilution was constant, the LNA-2 assay was duplexed with the *Gapdh* assay, and the copy number of *Kras^Lox-G12D^* was calculated by halving that of *Gapdh*. A good correlation between the actual and theoretical copy number was observed down to the tenth dilution, which corresponded to a copy number of 0.75 and allele frequency of 0.1% (Fig. 4E; Table S1). The dropout at the 11th dilution was consistent with only a 38% chance of a positive droplet being present, thus confirming the suitability of the LNA-2 assay for the sensitive detection of *Kras*^*Lox-G12D*^ in plasma.

### Cre-mediated recombination introduces mutations in the *Kras^Lox-G12D^* allele

Cre recombinase is known to have DNA-damaging activity (Loonstra et al., 2001), but its potential for introducing mutations at target recombination sites, within the *Kras^LSL-G12D^* allele, has not been investigated previously. To investigate whether possible sequence variation as a result of Cre expression may impact PCR sensitivity, DNA was extracted from a formalin-fixed paraffin-embedded section of *Kras^+/Lox-G12D^* mouse lung tissue containing multiple adenomas as determined by histological analysis (Fig. S5A). A 180-bp region surrounding the recombined LoxP sequence was PCR amplified and cloned, and 40 individual clones were sequenced. Sequence alignment revealed point mutations within 12 of the 40 clones, clustered within and around the *Sal*1 restriction sites (Fig. S5B,C). To confirm that these mutations were not induced by errors from the DNA polymerase during PCR amplification, a 140-bp wild-type (WT) region of the *Kras^WT^* allele co-amplified by PCR in the same reaction was also cloned and sequenced, but no mutations were identified (data not shown). Thus, Cre recombination of LoxP sequences introduces point mutations within target sequences that may have an impact on the efficiency of PCR amplification.

### Monitoring ctDNA by measuring *Kras^Lox-G12D^* levels in plasma

**Fig. 5.**
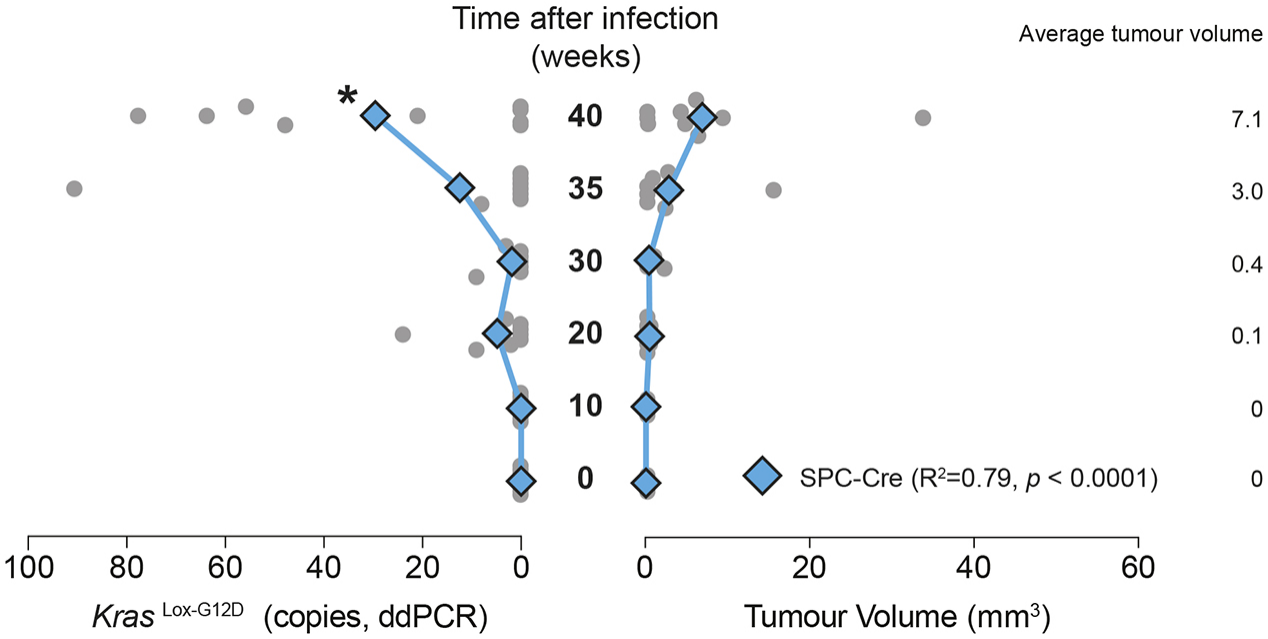
**Detection of the *Kras^Lox-G12D^* allele in plasma using ddPCR.** Analysis of *Kras^Lox-G12D^* levels in cfDNA by ddPCR and the total tumour burden in *Kras^+/LSL-G12D^* mice over a time course following infection with Ad5-mSPC-Cre. Mean values are indicated by diamonds/lines; values for individual mice are indicated by circles (*n*=8-9 at each time point). *P<0.05 for two-tailed unpaired Student's *t*-test comparisons between mean values at a given time point and values at time=0. The correlation coefficients between *Kras^Lox-G12D^* levels and tumour burden are indicated (linear regression analysis; *P*-value, goodness of fit). Datasets for total tumour volume are also used in Fig. 3.

We next applied the ddPCR assay developed above to monitor levels of the *Kras^Lox-G12D^* allele in the plasma ctDNA fraction of *Kras^+/LSL-G12D^* mice infected with Ad5-mSPC-Cre over a time course of 0 to 40 weeks. We chose to perform the analysis in Ad5-mSPC-Cre-induced mice only, because Ad5-CMV-Cre is known to induce recombination of the *Kras^LSL-G12D^* allele in non-tumour cells (Kamata et al., 2017). We observed a significant increase in the number of copies of the *Kras^Lox-G12D^* allele at 40 weeks p.i compared with samples at baseline, whereas an increase in tumour volume was evident from 35 weeks onwards using micro-CT (Fig. 5). There was some variability amongst different mice, with two of eight animals showing no detectable *Kras^Lox-G12D^* allele in blood at 40 weeks p.i. These same two mice were found to show low tumour volumes, as determined by micro-CT. Thus, although these data demonstrate that ctDNA can be detected in the circulation of *Kras^+/Lox-G12D^* mice at extended time points, changes in tumour volume are not reliably detected earlier by ctDNA than by micro-CT.

## DISCUSSION

The overall survival of cancer patients is greatly improved if the disease is diagnosed at an earlier stage, such as with CT screening in lung cancer (National Lung Screening Trial Research et al., 2013). However, the implementation of widespread CT screening in healthcare pathways is costly and impractical in many cases. Therefore, cheaper and more tractable alternatives are needed.

With regard to non-small cell lung cancer (NSCLC), extensive phylogenetic genomic sequencing in the TRACERx trial has identified evolutionary drivers of the disease (Jamal-Hanjani et al., 2017). Postoperative serial ctDNA profiling was shown to predict metastatic relapse in patients with NSCLC, and tumour volume, measured by CT volumetric analysis, correlated with the mean clonal plasma ctDNA variant allele frequency, becoming optimally detectable at volumes greater than 10 cm^3^ (Abbosh et al., 2017).

The application of ctDNA monitoring to the earlier detection of lung cancer has been more difficult due to the challenges associated with the identification of patients with pre-malignant lesions. For this reason, we modelled this in a well-characterised genetic mouse model of lung adenocarcinoma development driven by oncogenic KRAS (Jackson et al., 2001; Sutherland et al., 2014). One of the major strengths of the autochthonous *Kras^LSL-G12D^* mouse model is the ability to control tumour initiation by exposure to adenoviruses expressing Cre recombinase, coupled with the ability to follow subsequent tumour progression (Shaw et al., 2005). Mice develop numerous pulmonary lesions, which are predominantly of the papillary subtype, and these lesions include AAH, resembling putative precursors of human lung adenocarcinoma (Nikitin et al., 2004). These progress to small adenomas that enlarge over time, with some developing into overt adenocarcinomas over prolonged periods (Jackson et al., 2001). By monitoring the time from Cre induction, this model enables the investigation of mice with defined, early-stage lung lesions. We show, for the first time, that cfDNA/ctDNA is released into the blood in this model and that the cfDNA/ctDNA level correlates with tumour volume as determined by micro-CT. Furthermore, rising cfDNA levels and ctDNA were both detected without evidence of progression to overtly invasive adenocarcinomas, suggesting that pre-malignant lung lesions are able to release fragments of DNA into the circulation.

Using micro-CT, we were able to serially track tumour growth, and we show that this approach provides an accurate volumetric measurement of lesions larger than 0.5 mm^3^ by comparison to histological quantitation of tumour volumes (Fig. 1E). Contrast agents have been used previously in the *Kras^+/LSL-G12D^* mouse model to better differentiate tumours from the surrounding vasculature (Lalwani et al., 2013), and detection of smaller tumours has been enhanced using bioluminescence and fluorescence biomarkers (Rodriguez et al., 2014). The use of these additional approaches and/or the implementation of more advanced, high-resolution techniques – such as positron-emission tomography or magnetic resonance imaging – would potentially facilitate the detection of individual tumours with volumes below 0.5 mm^3^ in the mouse.

Detection of the *Kras^Lox-G12D^* allele in total cfDNA by PCR proved extremely challenging due to the palindromic nature of the LoxP sequence and sequence homology between the *Kras^WT^* and *Kras^Lox-G12D^* alleles. In addition, data from Fig. S5 show that Cre-mediated recombination introduces mutations at the recombination site, which potentially affect the performance of the assay. After multiple attempts, using several different approaches, a specific ddPCR assay was developed that was applied to plasma cfDNA samples. To our knowledge, this represents the first report of assays for detection of the Cre-recombined *Kras^Lox-G12D^* allele in *Kras^LSL-G12D^* mice.

The comparison between mice infected with Ad5-CMV-Cre and those infected with Ad5-mSPC-Cre shows that, at similar tumour burden, there are comparable total cfDNA levels in mice regardless of the adenovirus used (Fig. 3). This observation suggests that the infection of lung resident myeloid lineage cells and/or the development of bronchial lesions by Ad5-CMV-Cre does not significantly affect the release of cfDNA, but this requires further exploration.

Total levels of circulating cfDNA are known to increase in patients with advanced disease (Leon et al., 1977; Madhavan et al., 2014; Newman et al., 2014). Consistently, we observed an increase in the total cfDNA concentration in mice with progressive disease (Fig. 3). However, the relationship between tumour size and cfDNA concentration in mice was driven primarily by mice with significantly larger-than-average tumour burden. Notably, elevated cfDNA/ctDNA was detected in mice with at least 14 tumours larger than 0.5 mm^3^, combining to a total volume above 7 mm^3^. For a single mass, this is equivalent to a tumour with diameter ∼2.5 mm. Although, in humans, CT is able to detect lung nodules as small as 1 mm in diameter, the malignant potential of such lesions is unknown and follow-up scans are recommended to monitor progression of suspicious lesions (Rubin, 2015). Potentially, cfDNA/ctDNA profiling could be implemented at this stage to monitor the emergence of larger pre-malignant or, indeed, malignant lesions and thus avoid multiple CT scanning. However, it should be borne in mind that the volumes of mouse plasma analysed in the present study (∼50 µl) relative to total mouse blood volume (2 ml) are not achievable in humans (typically 2 ml from a total blood volume of 5 l) and therefore would require the development of more sensitive assays to profile the appropriate variant alleles.

In summary, we demonstrate the ability to detect the release of total cfDNA and tumour-derived ctDNA from mice bearing pre-malignant lung lesions with a total tumour volume in excess of 7.0 mm^3^. This discovery is potentially encouraging for the use of cfDNA/ctDNA profiling in the detection of pre-malignant lesions of the lung adenocarcinoma pathway in humans.

## MATERIALS AND METHODS

### Animal husbandry

Animal experiments were performed under the UK Home Office (HO) licence authority. Infected mice (C57BL6/J), both males and females, randomly selected for treatment, were analysed at experimental endpoints. Experimental animals underwent regulated procedures with a maximum severity classification of ‘moderate’ according to the HO guidelines*. Kras^+/LSL-G12D^* mice were genotyped according to the Jacks laboratory-recommended protocol (https://jacks-lab.mit.edu/protocols/genotyping/kras_cond). Intranasal inhalation of adenoviral vectors was performed using Ad5-CMV-Cre, Ad5-mSPC-Cre or Ad5-CMV-βgal in mice with an age range between 8 and 20 weeks. Mice were anaesthetised with 3% vapourised isofluorane in oxygen, in an induction chamber. The viral supernatant (50 µl) was loaded into the nasal aperture of each individual animal. Viral concentrations were 5×10^7^ plaque-forming units for Ad5-CMV-Cre and Ad5-CMV-βgal, and 1×10^8^ for Ad5-mSPC-Cre viruses. All packaged adenoviruses were purchased from the Viral Vector Core Facility (University of Iowa, Iowa City, IA, USA).

### Blood collection and plasma isolation

For longitudinal time points, blood was withdrawn from saphenous veins, whereas cardiac blood samples were taken at the endpoint. For mouse saphenous blood sampling, 40 μl was pipetted into 200 μl EDTA (4.5 mM, pH 8.0) in phosphate-buffered saline (PBS). For cardiac blood sampling, blood (typically, 200 µl) was collected from terminally anaesthetised mice into K_3_-EDTA vacutainers (BD Biosciences). Blood was centrifuged at 1000 ***g*** for 10 min, and the plasma supernatant was centrifuged at 1000 ***g*** for a further 10 min. Blood samples were processed promptly to reduce haemolysis; samples with evidence of haemolysis by visual inspection were excluded from further analysis. Plasma volumes were adjusted to 200 µl with PBS before extraction of cfDNA.

### Extraction of cfDNA

cfDNA was extracted from 200 µl plasma sample using a QIAamp DNA Blood Mini kit (Qiagen) and eluted in 50 µl TE buffer (10.0 mM Tris-HCl, 0.5 mM EDTA, pH 8.0). Purified DNA was stored at −20°C prior to use. Once extracted, the cfDNA concentration was measured using a Qubit 2.0 fluorometer with Qubit dsDNA HS Assay reagents (Thermo Fisher Scientific), with a detection range of ≥10 pg/µl.

### Micro-CT imaging

Lung and tumour volumes were quantified using a Quantum FX micro-CT Imaging System (PerkinElmer). Animals were anaesthetised using a continuous flow of vapourised isofluorane. Animals were imaged for 34 s using a field of view of 40 mm with respiratory gating. Each of these scans subjected the animal to ∼20 mGy radiation, a relatively low dose well within the range recommended to avoid irradiation artefacts over the course of the study. To determine volumes for identified tumours and whole lung volumes, Caliper micro-CT analysis software was used.

### Histological analysis of tumours

Lungs were resected and fixed in 4% paraformaldehyde for 24 h at room temperature. Tissue was then transferred to 70% ethanol and stored at 4°C. Tissue was embedded in paraffin, and 5 µm sections were cut using a microtome. For serial sectioning, sections at 100 µm intervals throughout the entire block were obtained. After sectioning, slides were stained with H&E as previously reported (Kamata et al., 2015). Stained slides were photographed using a Leica DM500 microscope and an ICC50 Camera (Leica), and overlapping images were merged using Adobe Photoshop (version 13.0.1.1). Slides were also scanned on a NanoZoomer-XR Digital slide scanner C12000 (Hamamatsu Photonics) and analysed using NDP.view2 software (Hamamatsu Photonics) to calculate tumour volumes.

### Cell culture

Mouse embryonic fibroblasts (MEFs) derived from *Kras^+/LSL-G12D^* mouse embryos were cultured and authenticated by PCR genotyping as previously described (Andreadi et al., 2012). MEFs were infected with Ad5-CMV-Cre at a multiplicity of infection of 500, and *Kras^+/Lox-G12D^* cells were grown by continuous culture. Genomic DNA from MEFs was extracted as previously described (Andreadi et al., 2012).

### PCR analysis

Primers used for qPCR analysis of *Gapdh* in cfDNA were as follows: forward 5′-CCTCACAATCTGTCTCACCTTATT-3′ and reverse 5′-GACCTCTGTAAGTCCGCTTTG-3′; a TaqMan probe with the sequence FAM-AGCCTTATTGTCCTCGGGCAT-BH1 was also used. For the *Kras^Lox-G12D^* assay, the following primers were used: forward 5′-CCAGTCAACAAAGAATACCGCAAGG-3′ and reverse 5′-TCTGCATAGTACGCTATACCCTGTG-3′; a TaqMan probe with the sequence HEX-TCGACATAACTTCGTATA-BH1 was also used. Underlined nucleotides represent LNA bases.

### qPCR

qPCR was performed as previously described (Rakhit et al., 2017) on the StepOnePlus Real-Time PCR System (Thermo Fisher Scientific) using TaqMan Fast Universal PCR Master Mix (Thermo Fisher Scientific). Thermal cycling conditions were as follows: 95°C for 10 min, followed by 40 cycles of 95°C for 15 s and 60°C (for the *Gapdh* assay) or 63°C (for the *Kras^Lox-G12D^* assay) for 20 s. Reactions were conducted in triplicate, including a no-template control and a positive control. *Gapdh* levels were adjusted for the plasma volume used for each cfDNA extraction.

### ddPCR

All reactions for ddPCR analysis were formulated as described previously (Rakhit et al., 2017). A QX200 Droplet Digital PCR System (Bio-Rad) was used, using the manufacturer's protocol and reagents. Thermal cycling conditions were as follows: 95°C for 10 min; 40 cycles of 95°C for 30 s and 63°C for 30 s; and 98°C for 10 min. The *Kras^Lox-G12D^* copies were adjusted for the plasma volume used for each cfDNA extraction. A no-template control and a positive control were included in every assay. Analysis was performed according to the manufacturer's instructions on QuantaSoft software (Bio-Rad).

### Statistical analysis

Statistical analysis was performed using GraphPad Prism 6 (www.graphpad.com). The data are presented as the mean value, and the error bars indicate ±s.d. or ±s.e.m. (as indicated). ****P<0.0001, ***P<0.001, **P<0.01, *P<0.05.

## Supplementary Material

Supplementary information
