# Supplementary material for: Early detection of pre-malignant lesions in a KRASG12D-driven mouse lung cancer model by monitoring circulating free DNA
Source: Dis Model Mech. 2019 Feb 12;12(2):dmm036863. doi: 10.1242/dmm.036863 (PMC6398498; doi:10.1242/dmm.036863)
Supplement: Supplementary information [file dmm-12-036863-s1.pdf]

SUPPLEMENTARY METHODS

Quantitative PCR with LNA bases

An alternative probe-based strategy was utilized in which a hydrolysis probe was designed to include so-called ‘locked nucleic acid’ (LNA) bases, a chemical modification that increases both the T_m and binding specificity of the probe while also providing strand-invasion properties (Suppl. Fig 2). This LNA probe, LNA-1, permitted PCR amplification of the recombined allele with a template of *Kras*$^{+/Lox\text{-}G12D}$ gDNA, but efficiency was poor (~ 85%). Moreover, generation of a standard curve of *Kras*$^{+/\text{Lox-G12D}}$ gDNA diluted into a background of *Kras*$^{+/LSL\text{-}G12D}$ (unrecombined allele) showed a complete dropout of the qPCR signal after the fourth dilution, corresponding to a recombined allele frequency of 6.25% (Suppl. Fig 3). Therefore, the LNA-1 probe strategy was unable to detect *Kras*$^{Lox\text{-}G12D}$ at low allele frequencies.

PCR amplification, cloning and sequencing of DNA of lung tissue

Sequence analysis of LoxP sequences following Cre-mediated recombination of the *Kras*$^{LSL\text{-}G12D}$ allele has not been reported previously. Since the assay for the detection of the Cre-Lox recombination requires the annealing of a probe to the LoxP sequence, mutations in and around LoxP may compromise assay performance. To investigate possible sequence variations, DNA was extracted from an FFPE core biopsy of *Kras*$^{+/Lox\text{-}G12D}$ mouse lung tissue, which was confirmed to contain multiple adenomas by haematoxylin and eosin histological analysis (Suppl. Fig 5A). A 180-bp region surrounding the recombined LoxP sequence was PCR amplified and cloned into a vector. Clones were screened by PCR, and 40 clones containing the 180-bp insert were sequenced. Sequence alignment revealed point mutations within 12 of

Disease Models & Mechanisms • Supplementary information

the 40 clones containing recombined LoxP (Suppl. Fig 5B). These mutations clustered within and around the *Sal*1 restriction sites and occurred in intronic sequences (Suppl. Fig 5C). Eleven of the 12 mutations were transitions (4 A>G; 1 G>A; 4 T>C; 2 C>T), and all mutations were confirmed by sequencing the opposite strand. To confirm that these mutations were not induced by errors from the DNA polymerase during PCR amplification of the insert sequence, the 140-bp WT region of *Kras* (co-amplified by the same primers) was also cloned. Forty clones were sequenced, and no mutations were identified (data not shown), thus confirming that the mutations observed following Cre-mediated recombination of LoxP sequences were induced by the recombination process.

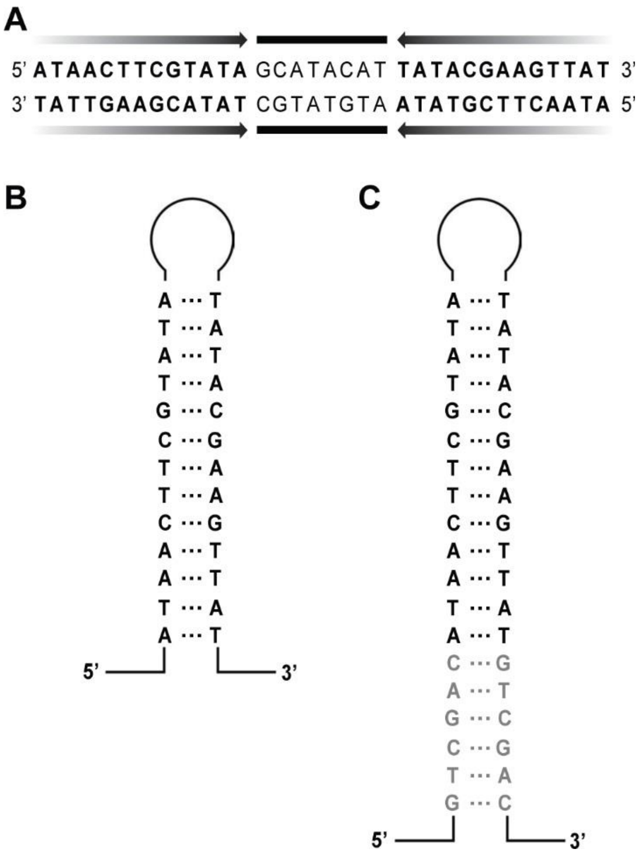

Figure S1. LoxP sequence and putative stem-loop structures.

(**A**) LoxP is a 34-bp sequence comprising two 13-bp palindromic sequences flanking a central 8-bp spacer sequence.

(**B**) LoxP forms a predicted 13-bp stem-loop structure with a melting temperature (T_m) of 55.6°C.

(**C**) In the *Kras*$^{LSL\text{-}G12D}$ allele, Cre-mediated recombination leads to the formation of a predicted 19-bp stem-loop structure with a T_m of 65.5°C. The 6-bp *Sal*1 restriction site is shown in grey, thus extending the stem-loop by 6 bp in the *Kras*$^{Lox\text{-}G12D}$ allele.

Disease Models & Mechanisms • Supplementary information

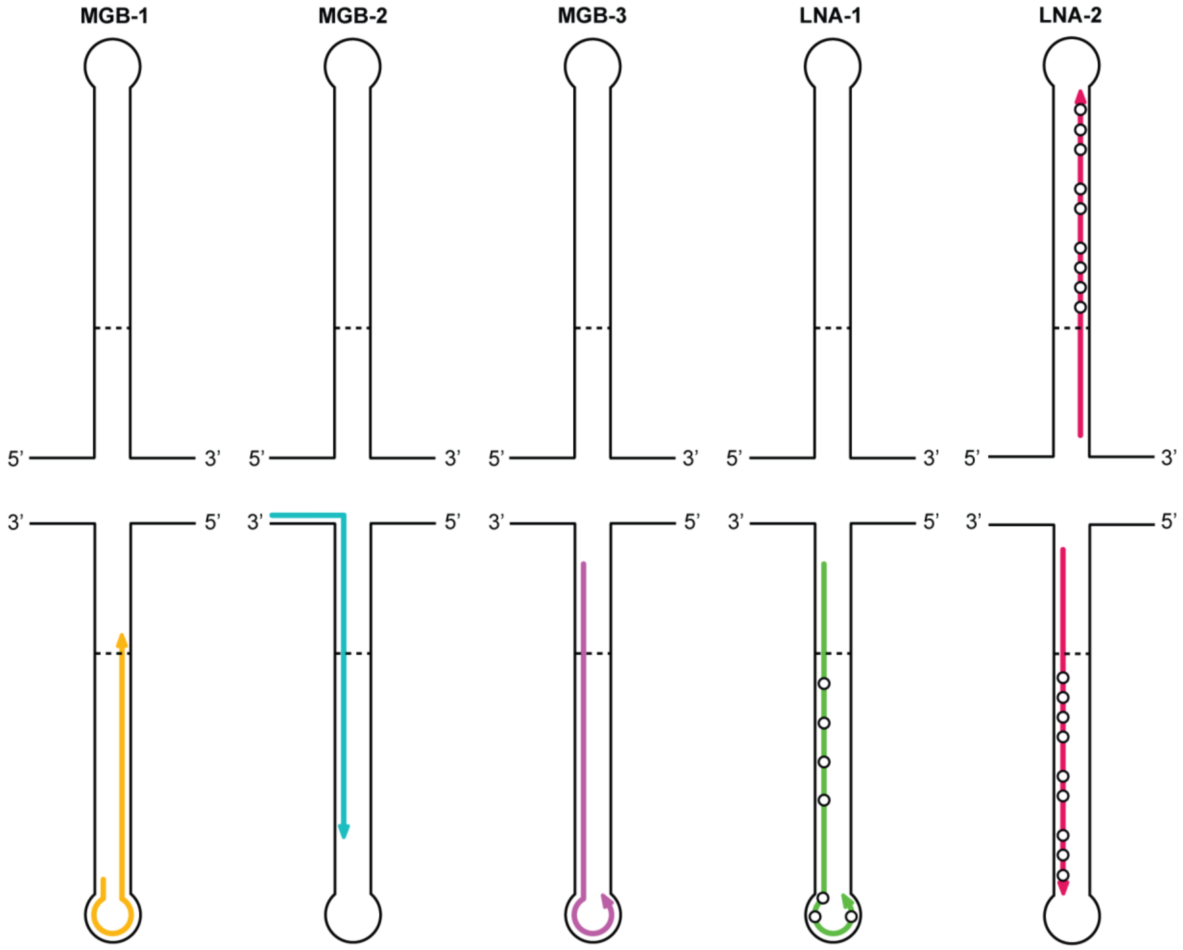

	MGB-1	MGB-2	MGB-3	LNA-1	LNA-2
**Tm (°C)**	70	74	72	70	74
**Length**	22	20	25	24	18
**LNA**	0	0	0	7	9
**Strand**	AS	AS	AS	AS	AS/S

Figure S2. Schematic diagram of the TaqMan and locked nucleic acid-modified probes targeting the recombined LoxP sequence in *Kras*$^{Lox\text{-}G12D}$.

Probes MGB1-3 are TaqMan 5’ FAM, 3’ MGB-labelled hydrolysis probes targeting various regions of LoxP/*Sal*1 on the antisense strand. Probes LNA1-2 are 5’ HEX, 3’ BHQ1-labelled probes with some bases ‘locked’ (black circles) using locked nucleic acid (LNA) technology. LNA-1 targets LoxP/*Sal*1 on the antisense strand only, whereas LNA-2 targets LoxP/*Sal*1 on both the sense and antisense strands.

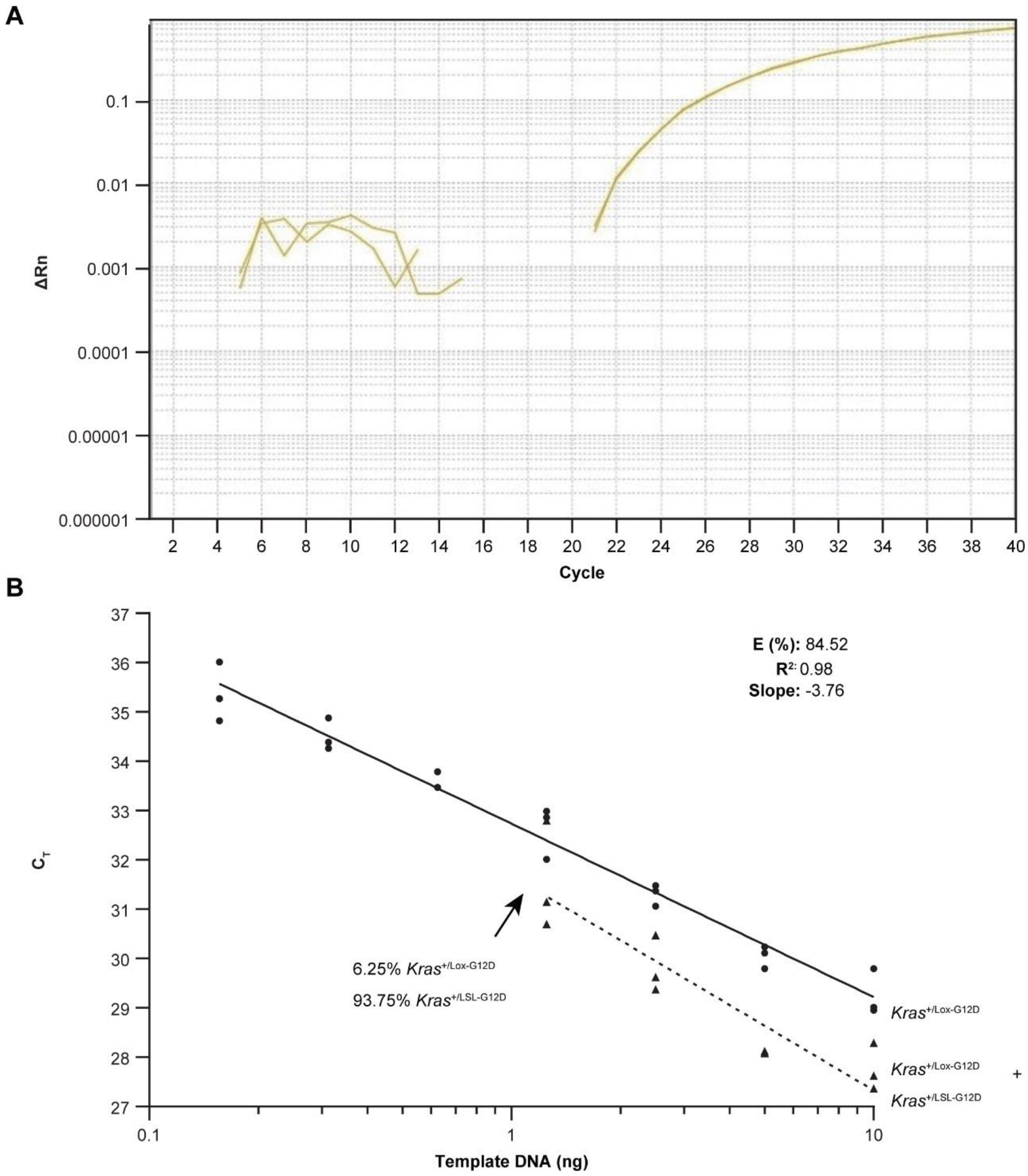

Figure S3. Performance of the 113-bp LNA-1 assay by qPCR.

(**A**) qPCR amplification plot of probe LNA-1 and primers flanking the recombined LoxP sequence, using a template of 100 ng of *Kras*$^{+/Lox\text{-}G12D}$ MEF gDNA.

(**B**) Performance of standard curves of serially diluted *Kras*$^{+/Lox\text{-}G12D}$ MEF gDNA in the absence (solid line) and presence (dashed line) of *Kras*$^{+/LSL\text{-}G12D}$ MEF gDNA. In a background of *Kras*$^{+/LSL\text{-}G12D}$ gDNA, the PCR signal was lost after the fourth dilution, corresponding to a *Kras*$^{Lox\text{-}G12D}$ frequency of 6.25%.

Disease Models & Mechanisms • Supplementary information

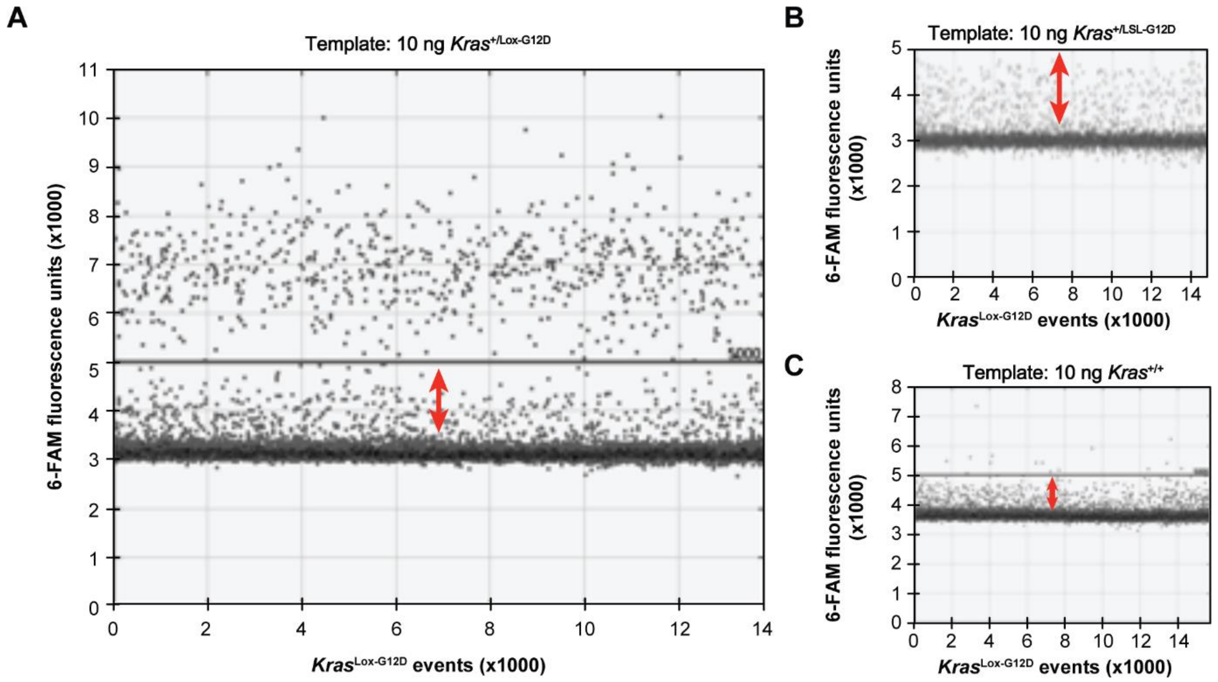

Figure S4. Performance of the 113-bp LNA-1 assay by ddPCR.

One-dimensional droplet plot of the 113-bp LNA-1 assay (comprising probe LNA-1 and primers flanking the recombined LoxP sequence in *Kras*$^{Lox\text{-}G12D}$) with (**A**) *Kras*$^{+/Lox\text{-}G12D}$ MEF gDNA; (**B**) *Kras*$^{+/LSL\text{-}G12D}$ MEF gDNA; and (**C**) *Kras*$^{+/+}$ MEF gDNA. A manual threshold of 5,000 fluorescence units was selected. Primers were annealed at 62°C, following a gradient PCR experiment to determine the optimum T_a (not shown). Red arrowed lines indicate areas of 'rain' caused by negative droplets above the main negative droplet clusters.

Disease Models & Mechanisms • Supplementary information

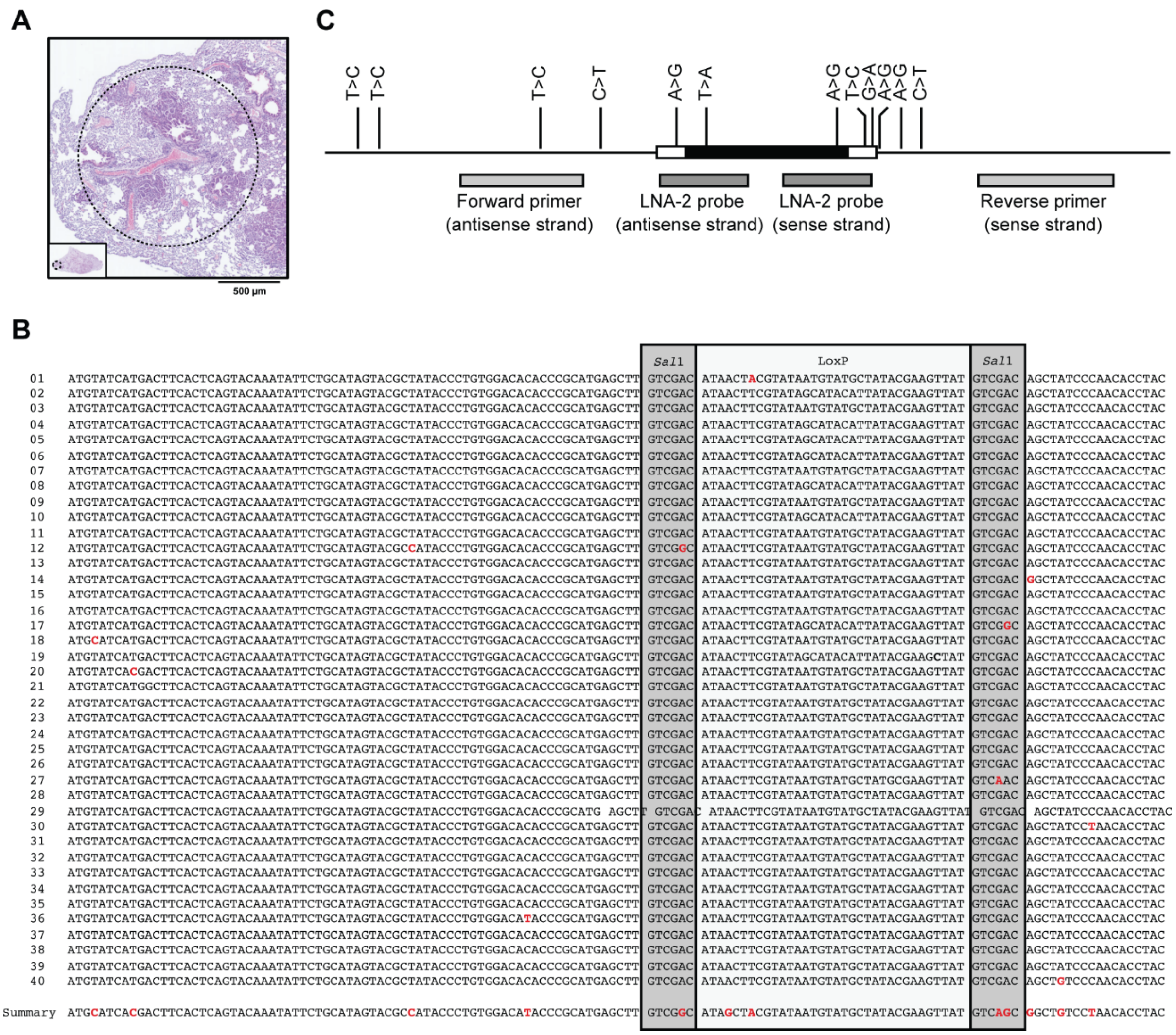

Figure S5. Assessment of sequence variation following Cre-mediated recombination

(**A**) H&E-stained section to indicate location of core biopsy from *Kras*$^{+/Lox\text{-}G12D}$ mouse lung. A 1.5-mm-diameter core biopsy was punched from a region of the *Kras*$^{+/Lox\text{-}G12D}$ mouse lung tissue, which was confirmed by H&E analysis to contain multiple adenomas.

(**B**) Alignment of reads for sequences within and around the recombined LoxP sequence in *Kras*$^{Lox\text{-}G12D}$; 140/180-bp primers flanking the recombined LoxP

sequence were used to amplify a 180-bp sequence in *Kras*$^{Lox\text{-}G12D}$. Mutations are shown in yellow. Primer sequences are not shown.

(**C**) A schematic diagram showing the position of mutations within and around the recombined LoxP sequence (black) in *Kras*$^{Lox\text{-}G12D}$. The grey boxes show the positions of the primers and LNA-2 probe in the 133-bp LNA-2 assay.

Disease Models & Mechanisms • Supplementary information

Table S1.

Theoretical copy number and frequency of *Kras*$^{Lox\text{-}G12D}$ in each serial dilution for the sensitivity analysis of the 133 bp LNA-2 assay

Serial dilution	*Kras*$^{+/Lox\text{-}G12D}$ gDNA (pg)	*Kras*$^{+/LSL\text{-}G12D}$ gDNA (pg)	*Kras*$^{Lox\text{-}G12D}$ copy number	*Kras*$^{Lox\text{-}G12D}$ frequency (%)
1	2500	5000	379	33.0
2	1250	5000	189	20.0
3	625	5000	95	11.0
4	312.5	5000	47	5.9
5	156.3	5000	24	3.0
6	78.1	5000	12	1.6
7	39.1	5000	6	0.8
8	19.5	5000	3	0.4
9	9.8	5000	1.5	0.2
10	4.9	5000	0.75	0.1
11	2.4	5000	0.38	0.05

Disease Models & Mechanisms • Supplementary information
